# Improving Time Estimation in Witness Memory

**DOI:** 10.3389/fpsyg.2019.01452

**Published:** 2019-06-27

**Authors:** Holly L. Gasper, Michael M. Roy, Heather D. Flowe

**Affiliations:** ^1^Elizabethtown College, Elizabethtown, PA, United States; ^2^School of Music, North-West University, Potchefstroom, South Africa; ^3^Centre for Applied Psychology, School of Psychology, University of Birmingham, Birmingham, United Kingdom

**Keywords:** witness memory, time estimation, anchoring, unpacking, bias

## Abstract

The present study sought to determine whether witness memory for duration could be improved. In three studies, we examined the effects of *unpacking* (breaking an event into its component parts), *anchoring* (supplying participants with a reference duration), and *summation* (summing component estimates). Participants watched a video-recorded mock crime and provided duration estimates for components of the crime (e.g., casing the car, unlocking the door, etc.) and for the total crime. Results indicate that bias in estimated duration was less for the sum of the parts than it was for the overall duration estimate. Further, the sum of the individual parts did not equal the total estimate, even though all estimates were given in sequence. Summing the component parts could be a more successful intervention than anchoring or unpacking and is easy to employ with witnesses.

## Introduction

Witness testimony plays an important role at every step of the criminal justice process. One important aspect of a crime that witnesses are frequently called on to remember is the duration of criminal events (e.g., the length of time that the perpetrator was in view of the witness; length of time that the crime lasted; Flowe et al., 2011). Time estimates for an event can help the criminal justice system, especially jurors and judges, to verify events and alibis (Loftus et al., 1987). Duration is also used as an indicator of likely memory accuracy. Among eyewitness memory experts, 81% agreed that events are less well remembered the less time a witness has to observe the event (Kassin et al., 2001). Indeed, duration can predict witness memory report accuracy. In a study of witnesses of armed bank robberies, robbery duration (as captured by video surveillance) was positively associated with the accuracy of offender descriptions given by witnesses (Fahsing et al., 2004). Yet, objective estimates of crime length are typically not available, and thus, investigations may have to rely on a witness’ estimates of duration. Research suggests that witnesses may often overestimate actual duration (Loftus and Doyle, 1987; Loftus et al., 1987) and that duration estimates can be influenced by wording, with shorter estimates given when actions are described in terms of quick movement (Burt and Popple, 1996). Research also suggests the longer a witness thinks about an event, the longer the estimate they may give (Zakay and Tsal, 1989). Further, juries do not seem to consider the fact that witnesses are often incorrect in their estimates of duration (Schmechel et al., 2006). Overall, research in the field of witness testimony indicates that estimates of crime duration may often be biased; but, unfortunately, this bias might not be recognized by the witness or the jury.

In support, reviews of studies in the time estimation literature indicate that most estimates of past task duration in general are inaccurate and easily biased (Wallace and Rabin, 1960; Fraisse, 1963; Ornstein, 1969; Poynter, 1989; Block and Zakay, 1997; Roy et al., 2005; Buehler et al., 2010; Halkjelsvik and Jørgensen, 2012). For example, task characteristics, such as whether the task is relatively short or long, can influence bias, with shorter tasks tending be overestimated and longer tasks tending to be underestimated (Bird, 1927; Yarmey, 2000; Roy and Christenfeld, 2008; Lejeune and Wearden, 2009; Tobin and Grondin, 2009). Given that bias exists in estimates of duration, many attempts have been made to reduce this bias using techniques such as anchoring (i.e., supplying participants with a reference duration; König, 2005; Furnham and Boo, 2011), unpacking (i.e., breaking an event into its component parts; Tversky and Koehler, 1994; Kruger and Evans, 2004) and summing (i.e., adding together the component parts; Forsyth and Burt, 2008). We examined whether or not anchoring, unpacking, and summing techniques that have been used in time estimation research can be helpful for decreasing the bias found in eyewitness estimates of crime duration.

### Anchoring

Research indicates that estimation can be improved with repeated experience with a task (Tobin and Grondin, 2012, 2015) and by being supplied with feedback for a new task or about a recent task (Roy et al., 2008). These improvements are likely due to participants being supplied with a frame of reference, or anchor (Furnham and Boo, 2011), before estimating task duration. In support, supplying participants with a single, common anchor can improve a number of different judgments (Bartoshuk et al., 2003), including estimates of duration (König, 2005; Furnham and Boo, 2011). Time estimation can also be influenced, both for better and for worse, by previously established anchors and knowledge about the task (Thomas et al., 2003, 2007; König, 2005; Thomas and Handley, 2008; Furnham and Boo, 2011; König et al., 2015). Anchoring can occur even when individuals are not prompted to use an anchor (Thomas and Handley, 2008). Roy et al. (2008) found that when participants were supplied with an average duration for a task, such as pumping gas or playing a video game, as an anchor, participants improved in their estimation of future task duration. Participants were able to correctly adjust their estimates upward or downward dependent upon whether they were likely to be faster or slower than average. Put another way, participants were good at estimating relative duration, whether something was slower or faster than another event, but bad at estimating absolute duration, how long the event took. Supplying a correct anchor, therefore, corrects participants’ absolute judgments (which tend to be incorrect) and allows them to make relative judgments (which tend to be correct). Here we attempted to improve estimation in witness memory by supplying participants with an anchor, a tone that they were told lasted 5 s, before estimating the duration of a crime that lasted approximately a half a minute as well as the subcomponents of that crime.

### Unpacking

Another technique that can be used to potentially improve estimation is unpacking, where an individual breaks down a task into subcomponent parts before estimating the total task duration (Tversky and Koehler, 1994; Kruger and Evans, 2004). It is possible that when people estimate the overall duration, they forget to include certain subcomponents or do not give those subcomponents proper weight when formulating their overall estimate. Making participants break down each section of the task can help to ensure that all portions of the task are included in the final estimation (Tversky and Koehler, 1994). Time estimation research has found that unpacking can, at times, lead to less biased estimations (Byram, 1997; Connolly and Dean, 1997; Jorgensen, 2004; Kruger and Evans, 2004; Tsai and Zhao, 2011; Min and Arkes, 2012; Hadjichristidis et al., 2014; Liu et al., 2014). For example, Kruger and Evans (2004) found that thinking about all the component parts before estimating duration for certain activities, such as preparing food and getting ready for a date, reduced bias. Research has also shown that more detailed descriptions can lead to more accurate evaluations of events overall (Van Boven and Epley, 2003). Here we examined whether estimating the duration of the subcomponents of a crime could decrease bias in the overall estimate of the crime duration.

### Summing

The unpacking manipulation also allowed us to examine the similar intervention of summing or segmentation (Macan, 1994; Forsyth and Burt, 2008). Whereas unpacking examines the impact of considering subcomponents on the overall estimation of duration, summing utilizes the combined estimations for the subcomponents in replacement of the overall estimation. However, the mechanism thought to result in an improved estimate is the same as unpacking – making sure that all subcomponents are considered and given the correct weight (Macan, 1994). In summing, all subcomponents are included, and it is not assumed, as in unpacking, that knowledge about those subcomponents will be transferred to the overall estimate. When the estimated duration for each of the subcomponents are added together, it can decrease bias in the estimated duration. For example, Forsyth and Burt (2008) found that bias in prediction for how long it would take to complete a series of office tasks was less when using the sum of the segments than when using the overall duration.

### Current Study

The present study sought to extend time estimation approaches to the field of eyewitness memory to assess whether time estimation for a crime could be improved. Specifically, anchoring, unpacking, and summing interventions were used to potentially aid eyewitnesses in estimating duration. Participants in three studies viewed a video of a crime, a theft from a car in Studies 1 and 2 and a theft from an electronics store in Study 3, with intervention varied before participants estimated the overall event duration. We sought to examine whether anchoring, unpacking, and summing interventions, either individually or in concert, could improve witness memory for crime duration.

## Study 1

### Methods

#### Participants

Sixty-six students at a small liberal-arts college in the United States (*n* = 59 females, *n* = 6 males, *n* = 1 no response; Age *M* = 18.95, *SD* = 2.53) participated in the study. All students were enrolled in a general psychology course at the college and participated for course credit. The Institutional Review Board at Elizabethtown College approved the research, and written informed consent was obtained from each participant prior to beginning the study. The study took less than 15 min to complete. Students were randomly assigned to one of four experimental conditions. Sample size for this initial study that explored extending time estimation interventions to the witness domain was determined by the number of participants that could be recruited over the course of one semester. It was expected that we would recruit about 60 participants, giving low power to detect interaction effects (∼15 per cell), medium power to examine main effects (∼30 per condition) and high power to examine within participants measures.

#### Design

The study was a 2 × 2 between-subjects design in which anchoring and unpacking were varied. For the anchoring manipulation, half the participants listened to a 5 s reference tone directly before estimating duration to supply them with a frame of reference. The other half of the participants were not given a reference tone. For the unpacking manipulation, half of the participants were asked to estimate the duration for each of the subcomponents before making an overall estimation. The other half of the participants supplied the total estimate first before estimating each of the subcomponents. Participants in both groups estimated all portions to be able to more directly compare their responses. Overall, there were four conditions: anchor-unpacking, no anchor-unpacking, anchor-no unpacking, and no anchor-no unpacking.

#### Materials and Procedure

Participants watched a 23 s video of a theft from a car (see Supplementary Material for link to video). The video shows a man walking up to a car with an open window, inspecting the inside of the car, and then unlocking and taking an item out of the car. The information bar, which shows elapsed time, was removed from the video, so participants could not access time information about the video. We chose this clip because it could easily be broken into discrete segments and because the total duration was not a value to which people would normally round. Previous research indicates that participants are overly reliant on rounded values (e.g., 30 s) when estimating duration and the use of tasks with durations that would be naturally rounded to could lead to inflated measures of accuracy (Huttenlocher et al., 1990; Forsyth and Burt, 2008). Participants were instructed to pay attention to the video because they would be answering questions about the video after it was finished; but, they were not told what aspects of the video the questions would be about. The video was broken down into segments in order of occurrence including how long it took the perpetrator to walk up to the car (3 s), how long it took the perpetrator to case/inspect the car (8 s), how long it took the perpetrator to unlock the car door (6 s), and how long it took the perpetrator to remove items from the car (4 s). The remaining 2 s were how long it took for the perpetrator to walk away from the scene; but, unfortunately, the surveys mistakenly did not include a question for the estimation of this component. After the participants watched the video, they either listened to a 5 s reference tone before estimating the duration (anchoring) or continued directly to estimating the duration. A tone was used because, intuitively, a short tone is an intervention that could be easily used in a number of different situations. A 5 s duration was chosen because it was approximately the average of the various segments. Participants were told to use the 5 s stimulus to help them in making their estimations of duration. The total time estimation was given either after estimating the duration for each of the components (unpacking), or first, before estimating the duration for the components. Estimates of other variables, such as perpetrator’s height and weight and how many visible cars were on the street, and confidence were also measured, but are not discussed here because they are not central to this study (see Supplementary Materials for analysis of these variables as well as the raw data for each study).

### Results

#### Descriptive Data

The data for the estimated duration for the segments of the video clip were positively skewed: walk up skewness = 1.21 [Kolmogorov–Smirnov’s *D*(66) = 0.22, *p* < 0.001]; casing the car skewness = 3.36, [*D*(66) = 0.25, *p* < 0.001]; unlocking the car door skewness = 1.27, [*D*(66) = 0.218, *p* < 0.001]; removing the items skewness = 6.23, [*D*(66) = 0.28, *p* < 0.001]; total estimate skewness = 2.24, [*D*(66) = 0.20, *p* < 0.001]. Due to the skew found in the estimated duration, descriptive statistics are reported in terms of median durations and the interquartile range (IQR). Participants estimated that it took 5 s (IQR = 5.00) to walk up to the car (actual duration = 3); 5 s (IQR = 3.25) to case the car (actual duration = 8); 2 s (IQR = 1.00) to unlock the car door (actual duration = 6); 2 s (IQR = 1.00) to remove items from the car (actual duration = 4); and that the total crime took 10 s (IQR = 13.00; actual duration = 23).

#### Bias

**FIGURE 1:**
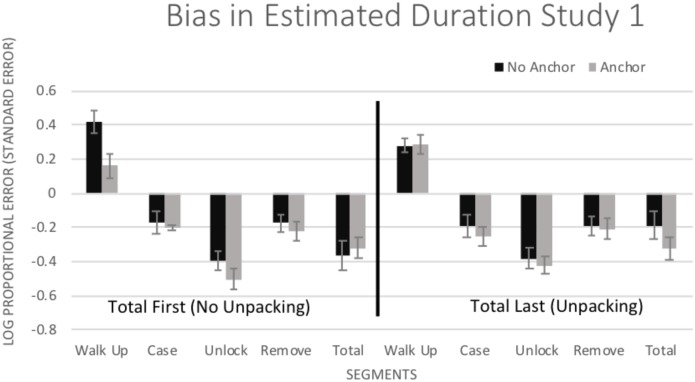
Mean (±1 *SE*) bias in estimated duration (log of estimated duration/actual duration) for all of the segments of the crime. Positive values indicate overestimation and negative values indicate underestimation.

To examine whether or not unpacking and anchoring helped to improve estimates, bias was examined in a 5 (Segment: individual segments and total) × 2 (Unpacking: total 1st or last) × 2 (Anchoring: anchor or no anchor) mixed-model ANOVA. Bias was measured by taking the log of the ratio of estimated duration divided by the actual duration (log proportional error; Roy and Christenfeld, 2007, 2008). This index helps to simplify interpretation; a negative score indicates underestimation, a score of zero indicates no bias, and a positive score indicates overestimation. The index also normalizes the data; there was, as reported, a strong positive skew in estimates of duration. In addition, this index allows comparison of bias in estimates across tasks of different lengths. There was a main effect of Segment [*F*(4,248) = 117.27, *p* < 0.001, η^2^ = 0.654], with differences in bias for the individual segments (see Figure 1). There was no significant main effect of Unpacking, *F*(1,62) = 0.18, *p* = 0.673, η^2^ = 0.003, or of Anchoring, *F*(1,62) = 2.22, *p* = 0.141, η^2^ = 0.035. There were no significant 2-way interactions *p*s > 0.45, η^2^s < 0.035. There was, however, a significant 3-way interaction for Segment × Unpacking × Anchoring, *F*(4,248) = 2.61, *p* = 0.013, η^2^ = 0.040. Simple effects tests (LSD) indicate bias within each individual segment was similar in size and direction regardless of condition, except there was less bias in estimation for walking up to the car after being presented with the anchor in the no unpacking condition (*p* = 0.014). It is unclear why anchoring would benefit the first judgment only after the total duration was estimated, but have no effect when directly following the anchor.

Central to our interests on the impact of Anchoring and Unpacking on estimated duration for the whole crime, neither anchoring nor unpacking improved the total estimate (*p*s > 0.22, η^2^s < 0.03). Participants’ estimations for the total duration were similar regardless of Anchoring and Unpacking conditions.

#### Sum

When participants estimated the different segments of the video clip, the sum of their estimations across the segments was larger than their estimate for the total duration (sum of segments = 14 s, total duration estimation = 10 s). It is possible that the sum of participants’ estimates for the individual segments was a better estimate of duration than was their total estimate. To further analyze this difference, a 2 (Total vs. Sum) × 2 (Unpacking) × 2 (Anchoring) ANOVA was conducted using bias (log proportional error). Sum is the addition of component parts for a total duration and Total is the estimate participants gave for the total crime. There was a significant main effect for Total vs. Sum, *F*(1,62) = 43.12, *p* < 0.001, η^2^ = 0.410, with the Sum being less biased than the Total. There were no significant main effects for Unpacking, *F*(1,62) = 0.34, *p* = 0.562, η^2^ = 0.005, or for Anchoring, *F*(1,62) = 1.15, *p* = 0.289, η^2^ = 0.018. There were also no significant two-way interactions (*p*s > 0.08, η^2^s < 0.05).

**FIGURE 2:**
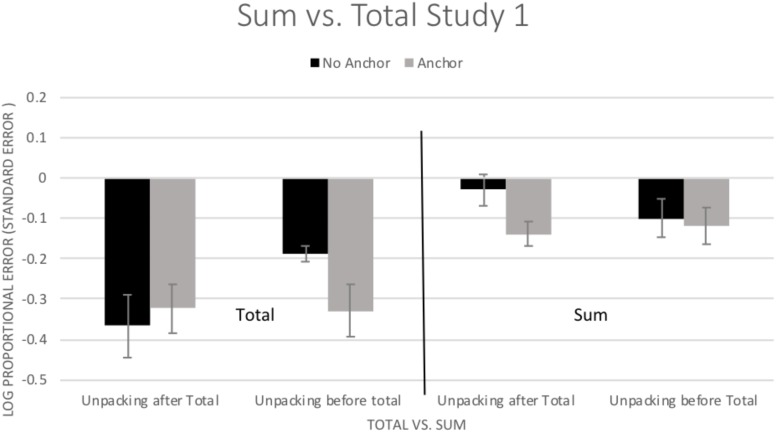
Mean (±1 *SE*) bias in estimated duration (log of estimated duration/actual duration) for the total duration estimation and for the sum of the segments. Positive values indicate overestimation and negative values indicate underestimation.

There was a significant three-way interaction of Total vs. Sum × Unpacking × Anchoring, *F*(1,62) = 4.80, *p* = 0.032, η^2^ = 0.072. As can been seen in Figure 2, the sum provided a less biased estimate of duration in all conditions. However, as indicated by simple effects tests (LSD), this improvement was not statistically significant in the no anchor-unpacking condition (*p* = 0.234), but was significant in the other three conditions (*p*s < 0.002). For the most part, the sum was superior to the total.

### Discussion

The total estimate was not improved by the anchoring and unpacking manipulations. However, results indicated that the sum of participants’ estimations for the component segments of the video were less biased than were participants’ estimates of the total duration of the video clip. Similarly, Forsyth and Burt (2008) found that when a *future* task was likely to be underestimated, using the sum of estimates for component parts decreased the tendency to underestimate. The results found here indicate that summing also helped decrease underestimation for *past* estimation of duration. The sum seemed to be better not because of a lack of bias in the estimates for the component parts, but due to the bias in estimating each of the individual parts canceling each other out when added together. One potential explanation for the pattern of bias found in the components might be due to their relative durations. In line with previous research (Bird, 1927; Yarmey, 2000; Roy and Christenfeld, 2008; Lejeune and Wearden, 2009; Tobin and Grondin, 2009), the shortest segment, walking up to the car, was overestimated, whereas the other portions were underestimated. To examine whether the result of this initial exploratory study could be replicated (Simmons et al., 2011; Cumming, 2014; Lakens and Evers, 2014), a follow-up study using the same materials was run with a larger, more diverse sample. The follow-up study also included a question for how long it took the perpetrator to walk away, which was not asked in Study 1.

## Study 2

### Methods

#### Participants

Participants were 160 Masters Workers from *Amazon Mechanical Turk* (65 = Females, 88 = Males, 1 = Other) ranging from 21 to 71 years of age (*M* = 38.28; *SD* = 10.59). Masters Workers are chosen by *Amazon Mechanical Turk (MTurk)* as highly regarded, reliable participants per *MTurk* statistics. Participants were paid $1.50 for participating in the study if their answers to an attention probe were accurate (confirming the content of the video). Six participants’ responses were removed from analysis for omitting or incorrectly answering the attention check question, leaving 154 total participants. Our goal was to have approximately 40 participants per condition (double the recommended 20 per cell of Simmons et al., 2011). The Institutional Review Board at Elizabethtown College approved the research, and electronic informed consent was obtained from each participant online prior to beginning the study (waiver of written consent approved by the IRB).

#### Materials and Procedure

*Qualtrics* was used to create an online study. Study 2 had the same design as Study 1, but also included a question asking participants to estimate how long it took the perpetrator to walk away from the scene. Participants were also asked to provide their own height and weight in addition to providing their age and gender (see Supplementary Material). Participants were randomly assigned to conditions through *Qualtrics* software.

### Results

#### Descriptive Data

Estimates of durations for component parts and totals tended to be positively skewed: Walk up skewness = 1.97 [*D*(151) = 0.22, *p* < 0.001]; Case car skewness = 3.61 [*D*(151) = 0.27, *p* < 0.001]; Unlock Car skewness = 2.76 [*D*(151) = 0.26, *p* < 0.001]; Remove item skewness = 1.61 [*D*(151) = 0.20, *p* < 0.001]; Walk away skewness = 3.46 [*D*(151) = 0.27, *p* < 0.001]; and Total crime skewness = 1.07 [*D*(151) = 0.13, *p* < 0.001]. In terms of median durations (the median and interquartile range (IQR) are reported due to skew), participants estimated that it took the perpetrator 5 s (IQR = 5.00) to walk up to the car (actual duration = 3); 5 s (IQR = 4.00) to case the car (actual duration = 8); 3 s (IQR = 3.00) to unlock the car door (actual duration = 6); 3 s (IQR = 3.00) to remove items from the car (actual duration = 4); 3 s (IQR = 2.00) to walk away from the scene (actual duration = 2); and that the total time was 20 s (IQR = 20.00; actual duration 23.00).

#### Bias

**FIGURE 3:**
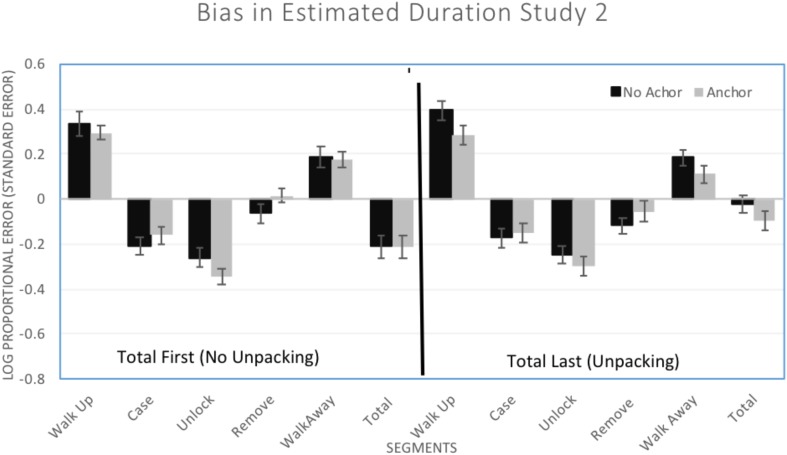
Mean (±1 *SE*) bias in estimated duration (log of estimated duration/actual duration) for all of the segments of the crime. Positive values indicate overestimation and negative values indicate underestimation.

To analyze whether or not Unpacking and Anchoring could improve estimation, bias (log proportional error) was examined in a 6 (Segments: individual segments and total) × 2 (Unpacking: total 1st or last) × 2 (Anchor: anchor or no anchor) mixed-model ANOVA. There was a main effect of Segments, *F*(5,730) = 216.25, *p* < 0.001, η^2^ = 0.597, with differences in bias for the individual segments (see Figure 3). There was no main effect of Unpacking, *F*(1,146) = 0.60, *p* = 0.441, η^2^ = 0.004, or of Anchoring, *F*(1,146) = 0.40, *p* = 0.526, η^2^ = 0.003, on bias. There was, however, a significant Segment × Unpacking interaction, *F*(5,730) = 5.56, *p* < 0.001, η^2^ = 0.037. Simple effects tests (LSD) indicate that the interaction was due to a significant difference in total time estimation of the crime with estimates less biased after Unpacking (*M* = -0.058, *SD* = 0.238) than before Unpacking (*M* = -0.211, *SD* = 0.307; *p* < 0.001). Bias was similar in size and direction for the individual segments with no differences due to the Unpacking manipulation. Unlike Study 1, Unpacking was successful in reducing bias for the total duration. Specifically, Unpacking reduced the tendency to underestimate found in the total duration.

There was also a significant Segment × Anchoring interaction, *F*(5,730) = 3.59, *p* = 0.003, η^2^ = 0.024. Simple effects tests (LSD) indicates that bias was similar in size and direction within each of the segments except that Anchoring improved estimation for the removing the item for the car segment. When estimating the time it took to remove the item from the car, there was a decrease in underestimation from *M* = -0.092 (*SD* = 0.235) when there was no anchor to *M* = -0.019 (*SD* = 0.233) when there was an anchor, *p* = 0.049. Overall, the Anchoring manipulation resulted in a small change in only one of the individual components.

There was not a significant Unpacking × Anchoring interaction, *F*(1,146) = 0.32, *p* = 0.572, η^2^ = 0.002, and no three-way interaction, *F*(5,730) = 0.44, *p* = 0.822, η^2^ = 0.003.

#### Sum

**FIGURE 4:**
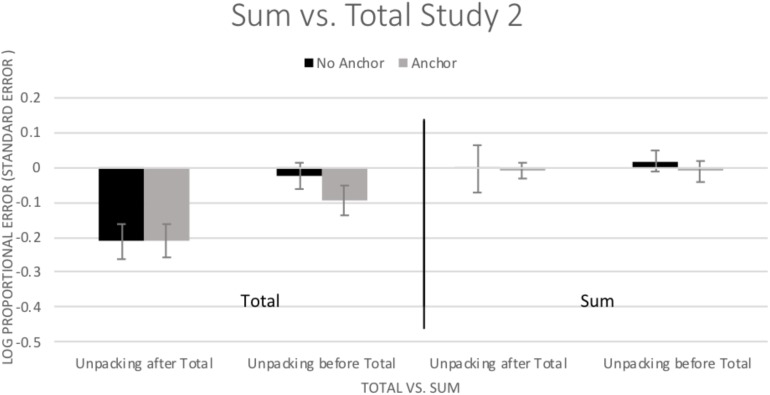
Mean (±1 *SE*) bias in estimated duration (log of estimated duration/actual duration) for the total duration estimation and for the sum of the segments. Positive values indicate overestimation and negative values indicate underestimation.

As with Study 1, participants’ estimations of the segments did not add up to the total duration estimate. For further analysis comparing the total crime and the sum estimate of the total crime, bias (log proportional error) was examined using a 2 (Total vs. Sum) × 2 (Unpacking) × 2 (Anchor) ANOVA. There was a significant main effect of Total vs. Sum, *F*(1,147) = 46.07, *p* < 0.001, η^2^ = 0.239, with the Sum being less biased than the Total (see Figure 4). There was also a main effect of Unpacking, *F*(1,147) = 6.03, *p* = 0.015, η^2^ = 0.039, with greater underestimation in the no unpacking condition compared to the unpacking condition. There was no main effect of Anchoring, *F*(1,147) = 0.61, *p* = 0.435, η^2^ = 0.004. The main effects of Total vs. Sum and Unpacking were qualified by a significant Total vs. Sum × Unpacking interaction, *F*(1,147) = 12.83, *p* < 0.001, η^2^ = 0.080. Simple effects tests (LSD) indicated that the Sum was always less biased than the total, but the amount of improvement depended on condition. In the no unpacking condition, the total (*M* = -0.211, *SD* = 0.281) was much more biased than the sum (*M* = -0.007, *SD* = 0.184), *p* < 0.001. In the unpacking condition, where bias was already reduced, the participants’ improvement from total (*M* = -0.056, *SD* = 0.242) to the sum (*M* = 0.005, *SD* = 0.185), *p* = 0.029 was smaller, but still significant. All other interactions were not significant (*p*s > 0.49, η^2^s < 0.003).

### Discussion

Here, using a larger and different sample than in Study 1, the main findings of Study 1 were replicated with the sum of component parts providing a less biased estimate of duration. There was a significant improvement in accurately estimating total duration due to unpacking, unlike Study 1, which found no effect. However, even when limiting to only the participants in the unpacking condition, in line with Study 1, the sum still outperformed the total estimate. Anchoring did not improve overall estimation with the anchor aiding estimation for only one subcomponent. Over the first two studies, results indicate that unpacking might at times be helpful. Nevertheless, the sum of components provided the least biased estimate (see also Forsyth and Burt, 2008). As with the previous study, bias was reduced for the sum because bias found in each of the individual component parts canceled each other out leading to less overall bias. Potentially, bias in the estimates might be explained by relative duration (e.g., Bird, 1927; Yarmey, 2000; Roy and Christenfeld, 2008; Lejeune and Wearden, 2009; Tobin and Grondin, 2009); here, the two shortest segments -approaching and walking away from the car - were overestimated in contrast to all of the longer segments which were underestimated.

However, both Study 1 and Study 2 used the same crime video and it is possible that the results were specific to that video. In Study 3, we examined the effect of unpacking and summing using a video of a different crime with a different overall duration.

## Study 3

### Methods

#### Participants

Ninety-Four Masters Workers from *Amazon Mechanical Turk* participated in this study. Participants were paid $1.25 for their participation if they correctly answered a probe question which asked them to identify the content of the video clip using a 4-item multiple-choice question. Seven responses were deleted because participants did not correctly identify the video clip as depicting a cellphone theft. The remaining 87 participants (33 = Female, 54 = Male) ranging from 21 to 62 (*M* = 33.72; *SD* = 7.85) years of age were included in analysis of the data. As with Study 1, we aimed at having around 40 participants per condition (Simmons et al., 2011). The Institutional Review Board at Elizabethtown College approved the research and electronic informed consent was obtained from each participant online prior to beginning the study (waiver of written consent approved by the IRB).

#### Design

This study focused on whether or not unpacking and summing were beneficial in estimating duration for eyewitness memory. Participants were randomly assigned through *Qualtrics* software to one of two conditions in which they either estimated the total crime duration before or after estimating durations for component parts of the crime (No Unpacking and Unpacking). Since the effect of anchoring was small and inconsistent in the previous two studies, it was dropped for this study.

#### Procedure

Participants watched a 34 s video clip of a cell phone theft from an electronics store with the information bar showing the elapsed time removed from the video. In the video, a man in an electronics store inspects a phone and a tablet before pocketing the phone and eventually leaving. As with the previous studies, the clip was chosen because it could be broken into discrete segments and because the overall duration was not one to which participants would normally round. Participants were instructed to pay attention to the video because they would be answering questions about the video after it was finished; but, they were not told what aspects of the video the questions would be about. The clip was broken down into four segments: the perpetrator looking at the phone (17 s), the perpetrator comparing the phone to a tablet (4 s), the perpetrator placing the phone into his pocket (4 s), and the perpetrator continuing to examine the tablet then walking away (9 s). After the clip, participants were prompted to answer a question regarding the content of the video. Then they continued to one of the two questionnaires estimating the total duration of the crime either before or after the durations of the four component parts were estimated. No other aspects of the crime, such as height and weight, were measured because we did not have data on their actual values.

### Results

#### Descriptive Data

As with Study 1 and 2, the estimates of duration were positively skewed: examining the phone skewness = 2.02 [*D*(87) = 0.23, *p* < 0.001]; comparing the phone with the tablet skewness = 3.73 [*D*(87) = 0.27, *p* < 0.001]; putting the phone in his pocket skewness = 5.96 [*D*(87) = 0.38, *p* < 0.001]; examining the tablet and walking away skewness = 3.80 [*D*(87) = 0.27, *p* < 0.001]; and total length skewness = 1.38 [*D*(87) = 0.21, *p* < 0.001]. In terms of median duration and interquartile range (IQR), participants estimated that it took 20 s (IQR = 19.00) to examine the phone (actual duration = 17); 6 s (IQR = 5.00 s) to compare the phone to the tablet (actual duration = 4); 2 s (IQR = 1.00) to put the phone in his pocket (actual duration = 4); 5 s (IQR = 4.00) to continue to look at the tablet and walk away (actual duration = 9); and 35 s (IQR = 30.00; actual duration = 34) for the total crime length.

#### Bias

**FIGURE 5:**
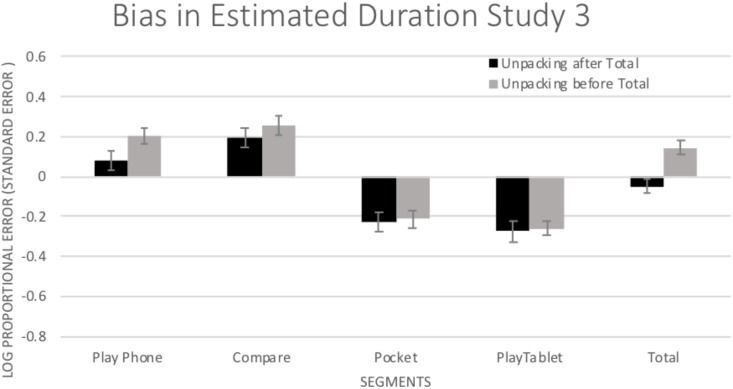
Mean (±1 *SE*) bias in estimated duration (log of estimated duration/actual duration) for all of the segments of the crime. Positive values indicate overestimation and negative values indicate underestimation.

To examine the potential benefits of unpacking, bias (log proportional error) was examined in a 5 (Segment: individual segments and total) × 2 (Unpacking: total 1st or last) ANOVA. There was a significant main effect of Segment, *F*(4,340) = 106.81, *p* < 0.001, η^2^ = 0.557, with differences in bias for the individual segments (see Figure 5). There was no main effect of Unpacking, *F*(1,85) = 2.92, *p* = 0.091, η^2^ = 0.033. There was a significant Segment × Unpacking interaction, [*F*(4,340) = 3.27, *p* = 0.012, η^2^ = 0.037]. Simple effects tests (LSD) revealed that bias was similar in size and direction for each of the individual segments regardless of condition except for the total duration. Opposite from Study 2, Unpacking here led to a *more biased* estimate for the total duration (*p* < 0.001). When participants did not Unpack before the total estimate, participants tended to slightly underestimate total durations (*M* = -0.048, *SD* = 0.236); but, when Unpacking occurred before the total time was estimated, participants greatly overestimated the total duration (*M* = 0.146, *SD* = 0.230). To put in terms of a percentage, participants underestimated the actual length of the crime by approximately 10% when they did not Unpack but overestimated by approximately 40% after Unpacking.

#### Sum

**FIGURE 6:**
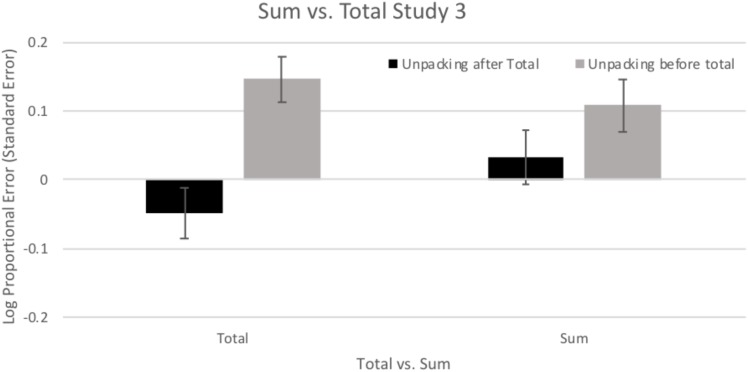
Mean (±1 *SE*) bias in estimated duration (log of estimated duration/actual duration) for the total duration estimation and for the sum of the segments. Positive values indicate overestimation and negative values indicate underestimation.

To examine potential benefits of Summing individual components, the total crime and Sum estimates of the Total crime were examined with a 2 (Total vs. Sum) × 2 (Unpacking) ANOVA of bias (log proportional error). There was not a significant main effect of Total vs. Sum, *F*(1,85) = 2.36, *p* = 0.128, η^2^ = 0.027. As can be seen in Figure 6, there was a significant main effect of Unpacking, *F*(1,85) = 7.08, *p* = 0.009, η^2^ = 0.077, with longer estimates for both the Sum and Total in the Unpacking condition. This was qualified by a significant interaction of Total vs. Sum × Unpacking, *F*(1,85) = 18.57, *p* < 0.001, η^2^ = 0.179. Simple effects tests (LSD) indicated that participants who were asked to estimate the total crime duration before Unpacking were less biased in their Sum of segments (*M* = 0.033, *SD* = 0.254) which reversed the tendency for underestimation found in their estimation of the Total crime duration (*M* = -0.048, *SD* = 0.236), *p* < 0.001. In contrast, participants in the Unpacking condition exhibited a reduction in the tendency to overestimate when using the Sum of segments (*M* = 0.108, *SD* = 0.255) compared to the Total estimation (*M* = 0.146, *SD* = 0.230), *p* = 0.044. Estimates of total duration varied greatly in direction and size of bias dependent on the use of Unpacking, but the Sum of the individual components was more consistent and less biased.

### Discussion

As in Study 1 and Study 2, results from Study 3 indicated that the sum of the component parts was less biased than were the estimates of the total duration. Here, unpacking made estimates of total duration worse, leading to pronounced overestimation of the actual duration. Unpacking tends to lead to longer estimates of duration, decreasing bias when underestimation is likely (as in Study 1), but leading to overestimation in all other cases (Connolly and Dean, 1997; Kruger and Evans, 2004; Tsai and Zhao, 2011; Min and Arkes, 2012; Hadjichristidis et al., 2014; Liu et al., 2014). It does not appear that unpacking can improve estimation in all cases, such as when overestimation is likely. It does appear, however, that summing the individual components can lead to better estimates regardless of condition. The improvement due to summing comes from the overestimation found for two of the segments canceling out the underestimation found for the other two segments.

Unlike the previous studies, bias in the estimates was not potentially related to relative duration in Study 3. The two segments that were underestimated were for pocketing the phone (4 s) and leaving the counter (9 s), with overestimation for examining the phone (17 s) and comparing the phone and tablet (4 s). The pattern of bias found here may have arisen from the excitement of the crime and the getaway, as interesting or exciting events are estimated to be shorter (e.g., Sackett et al., 2010), or the increased coherence of the event, as more coherent or predictable actions are judged as shorter (e.g., Boltz, 1998), or the amount of information remembered about the event, as events that have less component parts are estimated to be shorter (e.g., Ornstein, 1969). Potentially, the emotionality of the event could also have caused the distortion found here, however, negative emotion tends to lead to longer estimates of duration (Grondin, 2010), opposite of what was found here. Regardless of cause, the sum was less biased than the total even when the total was overestimated in one condition and underestimated in the other. Here results differ from those found by Forsyth and Burt (2008) when summing was used to shift bias in estimation for completion of a future task. They found that in all cases, summing led to longer estimates – decreasing underestimation, but *increasing* overestimation. We found that summing decreased bias both for underestimation and overestimation when estimating the duration of a previously viewed crime.

## General Discussion

Participants were consistently biased in their estimates of duration across three studies. Given this bias, it is worthwhile to examine ways in which estimation for the duration for a crime can be improved.

This research was designed to examine the effect of anchoring, unpacking and summing on estimates of total crime estimation. Results indicated that neither unpacking nor anchoring had the intended effect on total task duration. Even though the unpacking manipulation did decrease bias in the estimated total duration for Study 2, unpacking had no effect in Study 1, and led to an increase in bias in Study 3. Unpacking before giving the final estimate of duration does not appear to be a consistent way to decrease bias. Like previous research (Connolly and Dean, 1997; Kruger and Evans, 2004; Tsai and Zhao, 2011; Min and Arkes, 2012; Hadjichristidis et al., 2014; Liu et al., 2014), results here indicate that unpacking leads to longer, not better, estimates. Anchoring had no influence on the total task duration, even when used in concert with unpacking. Anchoring did at times influence the estimated duration for the segments, but the effect was small and inconsistent. We used a short reference tone as an anchor because it is a method that could easily be used in a number of situations. However, given that changes in modality of the task – audio or visual – is associated with differences in estimation (Wearden et al., 2006), it is possible that anchoring could be more beneficial if a different type of anchor was used. For example, a short video of a person performing a similar task, such as walking down the street, might be a better anchor than the short tone employed here because it is similar in modality and action to the target. The use of motion in the anchor might be important as research indicates that velocity of motion can influence estimation of duration, with faster movement associated with longer estimates of duration (e.g., Brown, 1995). Additionally, we used a stimulus that was similar in length to the subcomponents of that task. If the goal is to improve the overall task duration, it may have been more beneficial to use an anchor similar in duration to the total task. Anchors that are closer to the event being estimated lead to less biased estimates (Thomas et al., 2003, 2007; König, 2005; Thomas and Handley, 2008; Furnham and Boo, 2011; König et al., 2015).

In all three studies, the sum of the segments did not add up to the total estimate supplied by the participants. However, the sum of the segments was less biased than was the total estimate. This result suggests that asking for estimates of the segments of an event and then adding them together might supply a less biased representation of the total duration of the event (see also Forsyth and Burt, 2008). Importantly, the sum of segments consistently decreased bias over all three studies, using different samples, different crimes and regardless of whether participants tended to underestimate (Study 1 and 2) or overestimate (Study 3) duration. However, a reduction in bias when using the sum of segments was not due to there being less bias in the individual segments, but rather due to bias across the different segments canceling each other out when combined into a sum. It might be expected that employing estimates of shorter duration would lead to less bias than that found in the longer estimate for the total duration, since size of error should be proportional to the overall duration. Yet, bias found for the individual segments was not drastically different from that found in the estimates for the total duration. In relation, Weber’s fraction—the relationship between the magnitude of what is being estimated and the variability of the estimates—appears to be inconsistent for estimates of duration (Grondin, 2010). It seems that the direction, not the size of bias, is important here. In general, bias in time estimation appears to be unavoidable whether estimating for shorter or longer segments. Summing takes advantage of the fact that bias is likely to spread between over and under estimates, leading to less overall bias when summed. In a way, this technique is similar to dialectical bootstrapping (Herzog and Hertwig, 2009), where participants make repeated estimates for an event and then those estimates are averaged together to lessen the effect of error found in any one of the single estimates.

As indicated by the large positive skew found in the present studies and the majority of previous studies (Block and Zakay, 1997; Roy et al., 2005), individual estimates can be at times heavily biased. Even though the individual estimates were as likely to be skewed as the overall estimate was, summing mitigated the effect of skew found in any one single estimate. A potential extension of the effect of summing within person comes when there are multiple witnesses for a crime that all give duration estimates. In this case, it would also be advisable to average together the estimates of all the witnesses to avoid the sometimes-large errors found in the individual estimates (see also Davis-Stober et al., 2014).

We also found variability in bias direction, sometimes resulting in overestimation and sometimes underestimation. This is not altogether surprising given that bias is affected by a host of variables. For instance, time estimation bias is associated with the relative duration of the event being estimated, with longer events often leading to underestimation (e.g., Roy and Christenfeld, 2008). There is also evidence that excitement can influence estimation. When the actions of the perpetrator are more exciting, participants tend to provide shorter estimates (e.g., Sackett et al., 2010). Another key factor seems to be the coherence of the event, with more coherent events more likely to be underestimated (e.g., Boltz, 1998). As a final example, complexity of the target event affects duration estimates, with less complex events associated with shorter estimates of duration (e.g., Ornstein, 1969; Block and Zakay, 1997). These examples underscore the importance of finding procedures that enable individual biases to balance each other out, such as summation of the individual components to provide a better overall estimate of total crime duration.

Further research should be conducted to verify these results over different settings and time durations. Both video clips used in this study depicted a fairly short theft. It might be that the results found here only apply to similar crimes. Previous research indicates that task characteristics that influence bias, such as task duration (e.g., Roy and Christenfeld, 2008), tend to have a similar effect when applied to different time frames (e.g., seconds, minutes, and days), but it is not clear if this would also apply to crimes of greatly varying durations. Further, the stimuli used here were videos of a crime enactment, not a real situation experienced by a witness. It is likely that factors that participants experience when viewing the crime firsthand, such as fear, can also influence estimated duration (e.g., Grommet et al., 2011). For instance, in a study of novice skydivers, those who rated themselves as more fearful estimated that that the amount of time elapsed between the beginning and end of the skydive was longer (Campbell and Bryant, 2006). Similarly, in the eyewitness context, research participants who watched a video of a more stressful bank robbery overestimated its duration to a greater extent than those who watched a less stressful version of the crime (Loftus et al., 1987). Also, estimations of duration in the present study were elicited fairly soon after viewing the crime, potentially unlike the majority of witness recall situations (Flowe et al., 2011). Length of delay between the critical event and estimating duration can influence the size and direction estimation bias, with longer delays often found to increase bias (e.g., Roy et al., 2008). Future research should, therefore, vary the length of delay between the crime and when it is recalled to test its effects on time estimation. Finally, the events here were segmented by the experimenters to better compare individual results. It might be that letting participants segment the task on their own would be more beneficial because this process also aids in overall recall for the event (e.g., Flores et al., 2017; Gold et al., 2017).

Though more research is necessary, the practical implication from these three studies is that bias can be decreased by having witnesses estimate the duration for each segment of a crime and then adding together those segments. Our results suggest that summation of crime segments can be an effective intervention that is also fairly easy to use and could lead to other potential benefits, such as prolonged memory for the event (Flores et al., 2017; Gold et al., 2017).

## Ethics Statement

This research was approved by the Elizabethtown College IRB. Informed consent was obtained from each participant prior to beginning the study.

## Author Contributions

All authors conceived and designed the studies, analyzed and interpreted the data, and contributed to the manuscript writing with the HG supplying the initial draft. HG collected the data.

## Conflict of Interest Statement

The authors declare that the research was conducted in the absence of any commercial or financial relationships that could be construed as a potential conflict of interest.
