# Supplementary material for: Improving Time Estimation in Witness Memory
Source: Front Psychol. 2019 Jun 27;10:1452. doi: 10.3389/fpsyg.2019.01452 (PMC6621925; doi:10.3389/fpsyg.2019.01452)
Supplement: Supplementary file 1 [file Presentation_1.pdf]

Supplemental Material

Link to video for Studies 1 and 2:

https://www.youtube.com/embed/mmLg3FhGh5s?modestbranding=1&controls=0

Study 1

Estimates for height, weight and number of cars were not expected to be influenced by the manipulations. Participants were asked to state the level of confidence they had for each question that was answered using a 9-point scale in which 1 was 'not at all confident', 5 was 'somewhat confident', and 9 was 'very confident'.

Other Measures

As a comparison to time estimation, we also examined bias (log proportional error) in judgments of height, weight and number of cars visible. For all three variables, a 2(Anchoring) x 2 (Unpacking) ANOVAs revealed only a significant main effect of unpacking on height, $F(1,62) = 6.31$, $p = .015$, $\eta^2 = .092$: Participants estimating total duration first tended to supply higher estimates for height ($M = 72.14$, $SD = 1.51$) than did participants estimating the total duration last ($M = 71.11$, $SD = 1.81$). All other results for height ($ps > .23$, $\eta^2s < .03$), weight ($ps > .77$, $\eta^2s < .01$), and number of cars ($ps > .73$, $\eta^2s < .01$) were not significant. Participants underestimated the height of the perpetrator ($M = -.008$, $SD = .011$, $t(65) = -5.78$, $p < .001$, $d = -.73$, Median = 72 in, Actual = 72.8), but overestimated perpetrator's weight ($M = .059$, $SD = .045$, $t(65) = 4.91$, $p < .001$, $d = 1.33$, Median = 190 lb, Actual = 165.4), and the number of cars visible ($M = .062$, $SD = .190$, $t(65) = 2.638$, $p = .010$, d = .32, Median = 5, Actual = 4). It should be noted that though participants were biased in their estimations for height, weight and number of cars, bias for these factors was much smaller than the bias found in the estimates for duration. There were no gender differences in bias for any of the variables, including duration ($ps > .13$).

Confidence

Participants' confidence in their estimates was compared using an 8 (item being estimated) x 2 (Unpacking) x 2 (Anchoring) mixed-model ANOVA. There was a significant effect of item, with confidence varying dependent on what was being estimated, $F(7,434) = 14.34$, $p < .001$, $\eta^2 = .19$. As can be seen in the mean values reported in Table 1, post hoc analyses (LSD) indicated that confidence ratings could be placed into 3 somewhat equivalent groups (estimates in those groups were not significantly different from each other, but significantly different from the estimates in the other groups). Receiving the highest confidence ratings was a group that included duration estimation for unlocking the door, removing the item and the total duration for the crime. Participants were somewhat less confident in their ratings of duration for walking up to the car, casing the car and for the height of the perpetrator. The least amount of confidence in ratings was reserved for the weight of the perpetrator and the number of visible cars. It is notable that participants were least confident for ratings that tended to have the smallest average bias. Participants were most biased in their estimations of duration, but also most confident. Confidence was not influenced by either of the manipulations, anchoring and unpacking, ps > .25, η^2s < .02.

Table 1

Study 1 Item	Mean Confidence Rating (St Dev)
Walking Up Duration	4.92 (1.32)
Casing Car Duration	5.00 (1.48)
Unlocking Door Duration	5.44 (1.61)
Removing Item Duration	5.61 (1.56)

Total Duration	5.44 (1.46)
Height of Perpetrator	4.97 (1.47)
Weight of Perpetrator	4.35 (1.55)
Visible Cars	3.94 (1.68)

The average accuracy of the time estimates was collapsed across segment and participants and plotted as a function of confidence and anchor. As can be seen in Figure 1, accuracy increased with confidence when an anchor was given, whereas there was no relationship between confidence and accuracy when no anchor was given. However, the error bars are overlapping, indicating no significant differences.

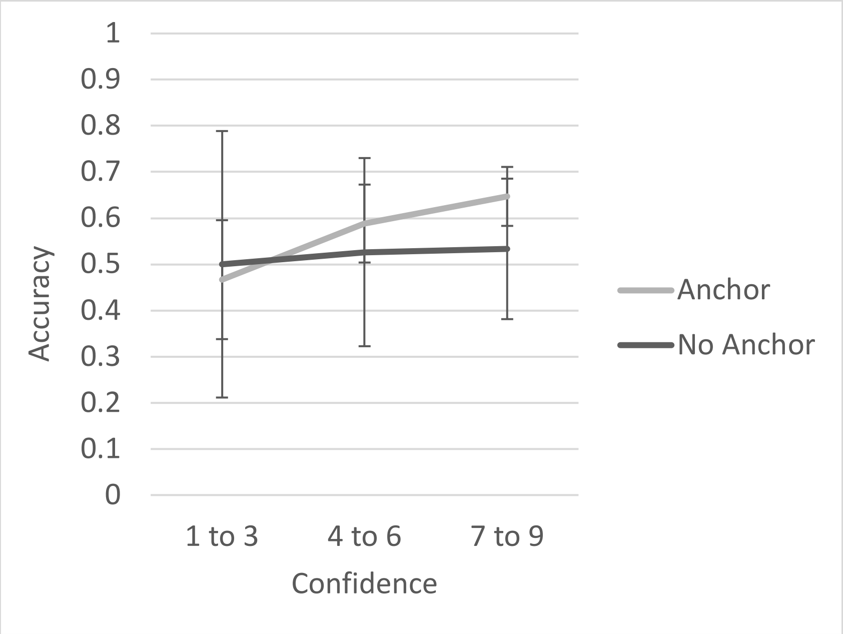

Figure 1. Average accuracy (±2 *SE*) of time estimates as a function of confidence level by anchor condition.

Study 2

Participants' height and weight were recorded to examine whether or not these factors influenced judgments of the perpetrator's height and weight (anchors can influence judgments such as height, Mussweiler & Strack, 2000, and weight, Frederick & Mochon, 2012).

Other Measures

We examined bias (log proportional error) in judgments of height, weight and number of cars visible. There was no influence of unpacking or anchoring on judgments of height, weight or number of cars (ps > .09, η^2s < .02). As in Study 1, participants underestimated the height of the perpetrator (M = -.010, SD = .012, t(153) = -10.07, p < .001, d = -.81, Median = 71 in, Actual = 72.8), overestimated the perpetrator's weight (M = .031, SD = .053, t(153) = 7.25, p < .001, d = .58, Median = 180 lb, Actual = 165.4), but, unlike in Study 1, exhibited no bias in estimation for the number of cars visible (M = .023, SD = .170, t(153) = 1.65, p = .101, d = .13, Median = 4, Actual = 4). As with Study 1, the bias found in the estimates of height, weight and number of cars was much smaller than the bias found in the estimates of duration.

There were no gender differences in bias for the variables measured here (ps > .17). Though height of the participant was not related to their estimation of perpetrator's height, r(150) = .037, p = .652, there was a small correlation between participant's weight and the estimation of the perpetrator's weight, r(150) = .210, p = .010. It is possible that participants used their own weight as an anchor for their judgment of perpetrator's weight.

Confidence

Confidence for the estimates was compared using a 9(item being estimated) x 2 (Unpacking) x 2 (Anchoring) mixed-model ANOVA. There was a significant effect of item, with confidence varying dependent on what was being estimated, F(8,1136) = 46.90, p < .001, η^2 = .25 (see Table 2). However, ratings of confidence for duration estimates were influenced by the

unpacking manipulation as indicated by a significant item by unpacking interaction, $F(8,1136) = 5.79$, $p < .001$, $\eta^2 = .04$. Simple effects tests (LSD) indicate that when the individual segments were estimated before the total, participants were more confident in their estimations for the walking up, unlocking the door, removing the item and walking away segments (ps < .04), but not more confident in their other estimations (ps > .07). Further, for both orders of estimation, participants were most confident in their estimations for the unlocking the door, removing the item and walking away segments, least confident for their estimation of the number of cars, with other estimations of confidence falling between these two groups. None of the other main effects or interactions were significant, ps > .13, η^2s < .02.

Table 2

	Mean Confidence (St Dev)	
Study 2 Item	Total First	Total Last
Walking Up Duration	3.79 (1.15)	4.54 (1.24)
Casing Car Duration	4.08 (1.30)	4.46 (1.29)
Unlocking Door Duration	4.51 (1.42)	5.00 (1.38)
Removing Item Duration	4.48 (1.31)	5.00 (1.25)
Walking Away	4.39 (1.32)	4.99 (1.35)
Total Duration	4.24 (1.45)	4.39 (1.29)
Height of Perpetrator	3.87 (1.33)	3.77 (1.42)
Weight of Perpetrator	3.81 (1.22)	3.58 (1.37)
Visible Cars	3.09 (1.55)	3.23 (1.70)

The average accuracy of the time estimates was collapsed across segment and participants and plotted as a function of confidence and unpacking condition. As can be seen in Figure 2, accuracy increased with confidence when total was given last (in the unpacking condition) compared to when the total was given first (in the no unpacking condition). However, the error bars are overlapping suggesting no difference in confidence accuracy calibration across conditions.

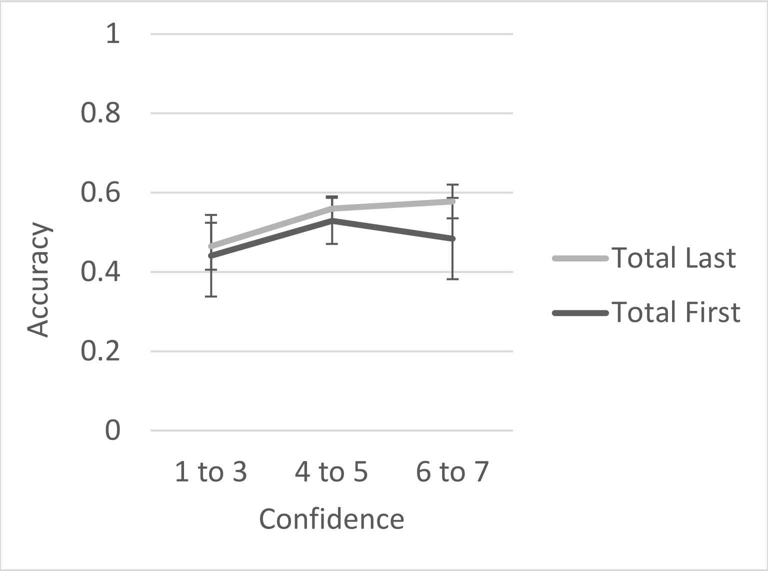

Figure 2. Average accuracy (±2 *SE*) of time estimates as a function of confidence level for the unpacking (total given last) and no unpacking (total given first) conditions.

Study 3

There were no gender differences in estimations of duration (*p*s > .11).

Confidence

Confidence in the estimates of duration was compared using a 5 (event being estimated) x 2 (Unpacking) mixed-model ANOVA. There was a significant effect of event, with confidence varying dependent on what was being estimated, $F(4,340) = 8.50$, $p < .001$, $\eta^2 = .09$. Post hoc analyses indicated that confidence was lowest for duration of the last segment of the video,

examining the tablet and leaving, but somewhat equivalently higher for all other aspects (see Table 3). Confidence was not influenced by the unpacking manipulation, *p*s > .27, η^2s < .02.

Table 3

Study 3 Item	Mean Confidence Rating (St Dev)
Examining Phone	4.33 (1.44)
Comparing Phone and Tablet	4.44 (1.47)
Pocketing Phone	4.94 (1.50)
Examining Tablet and Leaving	4.16 (1.45)
Total Duration	4.31 (1.41)

The average accuracy of the time estimates was collapsed across segment and participants and plotted as a function of confidence and unpacking condition. As can be seen in Figure 3, accuracy increased with confidence in both unpacking conditions. There was no significant difference in confidence-accuracy calibration as evident by the overlapping error bars.

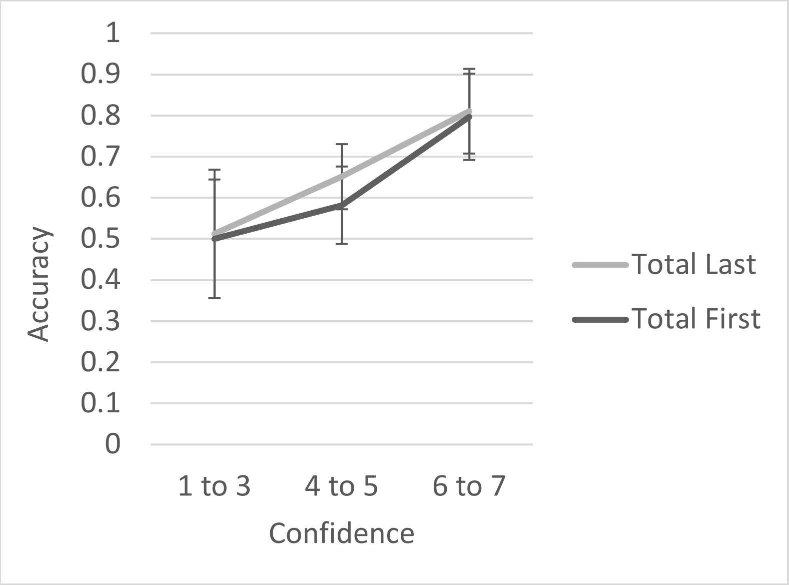

Figure 3. Average accuracy (±2 *SE*) of time estimates as a function of confidence level for the unpacking (total given last) and no unpacking (total given first) conditions.

Unpacking in Study 2 and anchoring in Study 1 tended to increase confidence-accuracy calibration, although the differences were not statistically significant. In Study 3, accuracy increased with confidence, regardless of unpacking condition.

References

Frederick, S. W., & Mochon, D. (2012). A scale distortion theory of anchoring. *Journal of Experimental Psychology: General*, *141*, 124.

Mussweiler, T., & Strack, F. (2000). Numeric judgments under uncertainty: The role of knowledge in anchoring. *Journal of Experimental Social Psychology*, *36*, 495-518.
